# Statins and Risk of Lower Limb Revision Surgery: The Influence of Differences in Study Design Using Electronic Health Records From the United Kingdom and Denmark

**DOI:** 10.1093/aje/kwv311

**Published:** 2016-06-16

**Authors:** Arief Lalmohamed, Tjeerd P. van Staa, Peter Vestergaard, Hubertus G. M. Leufkens, Anthonius de Boer, Pieter Emans, Cyrus Cooper, Frank de Vries

**Keywords:** arthroplasty, case-control studies, cohort studies, hydroxymethylglutaryl-CoA reductase inhibitors, pharmacoepidemiology

## Abstract

Previous observational studies on statins have shown variable results based on the methodology used. Our objective was to study the association between statins and orthopedic implant failure and to explore the influence of methodological differences in study design. Our study base consisted of patients with a primary total joint replacement in Denmark and the United Kingdom (*n* = 189,286; 1987–2012). We used 4 study designs: 1) case-control (each patient with revision surgery matched to 4 controls), 2) time-dependent cohort (postoperative statin use as a time-varying exposure variable), 3) immortal time cohort (misclassifying the time postoperatively before statin use), and 4) time-exclusion cohort (excluding the time postoperatively before statin use). Cox proportional hazards models and logistic regression were used to estimate incidence rate ratios. In the time-dependent cohort design, statin use was associated with a decreased risk of revision surgery (adjusted incidence rate ratio (IRR) = 0.90, 95% confidence interval (CI): 0.85, 0.96), which was similar to our case-control results (IRR = 0.87, 95% CI: 0.81, 0.93). In contrast, both time-fixed cohort designs yielded substantially lower risk estimates (IRR = 0.36 (95% CI: 0.34, 0.38) and IRR = 0.65 (95% CI: 0.63, 0.68), respectively). We discourage the use of time-fixed cohort studies, which may falsely suggest protective effects. The simple choice of how to classify exposure can substantially change results from biologically plausible to implausible.

Primary total joint replacements (TJRs) of the hip and knee substantially alleviate pain and improve physical function and quality of life in patients with moderate to severe osteoarthritis (1). Each year, approximately 1.8 million of these procedures are performed worldwide (2, 3). Up to 8.3% of recipients need their joint implants revised in the first 10 years (4), and these revisions are associated with poorer clinical outcomes compared with primary TJR (5, 6).

Observational studies have suggested many beneficial pleiotropic effects of statins, including a reduced risk of fracture and cancer (7, 8). Similarly, statins have been proposed to prevent implant revision failure (9). In a Danish case-control study, Thillemann et al. (9) reported a 66% reduction in implant revision with statin use, although they could not find a dose-response relationship.

These potential beneficial effects could be explained on a biological etiological basis (10–12). However, the seemingly beneficial effects from observational studies could also be explained by study design and analytical choices. Previous studies on statins have shown discrepant results when the data were analyzed for a second time in the exact same database. For example, in the first British observational study, Meier et al. (7) used the General Practice Research Database (GPRD) to examine the risk of fracture with statin use and reported an odds ratio of 0.55 (95% confidence interval (CI): 0.44, 0.69). In contrast, in the second study using the GPRD, van Staa et al. (13) did not find such a protective effect. In a third GPRD study, examining the reasons for the discrepant results, de Vries et al. (14) found that the age band for matching cases and controls, the selection of potential confounders, the exclusion of high-risk patients, and different definitions for exposure time windows could explain the different results between the first 2 GPRD studies. Similarly, a British cohort study on bisphosphonates showed a 46% reduction in joint implant failure (15), whereas a 34% detrimental trend was found in a Danish case-control study (16).

These reanalyses have taught us that arbitrary decisions in observational studies may have a large impact on the study results and need to be explored in great detail. Some of the microdecisions that have been hypothesized to influence study results include: 1) use of case-control designs versus cohort designs, 2) use of time-dependent cohort designs versus time-fixed cohort designs, 3) selection of confounders, 4) techniques for dealing with confounding (including propensity score analyses), and 5) selection of the data source (e.g., hospitalization registries or general practice–based electronic health records) (14, 17, 18). As Suissa et al. (17) demonstrated, we can expect a large impact from the exposure classification (i.e., time-dependent vs. time-fixed cohort designs). Given the increase in popularity of the time-fixed cohort design—in particular when evaluating the protective effect of medications on implant failure (15)—we sought to further build on these findings in the present study. In contrast, we expected no substantial difference between the various methods to deal with confounding using electronic health records (despite major efforts to optimize these techniques). The confounding data in electronic health records are often limited by the quality of the data itself, but this has not been studied comprehensively before.

The objectives of this study were 1) to evaluate the association between statins and implant failure in patients with primary TJR surgery, 2) to study the impact of differences in study design, and 3) to assess the influence of using 2 different data sources.

## METHODS

### Source population

We conducted a retrospective multicountry study using data from the Clinical Practice Research Datalink (CPRD), previously known as the GPRD, and the Danish National Health System (DNHS). The CPRD collates the computerized medical records of general practitioners. The data recorded in the CPRD since 1987 include demographic information, prescription details, clinical events, preventive care provided, specialist referrals, hospital admissions, and major outcomes (https://www.CPRD.com). The DNHS keeps computerized medical records on all contacts with hospitals and general practitioners and on income, degree of education, working status, civil status, migrations, the use of medications, and causes of death for the entire Danish population (5.5 million inhabitants). In both data sources, data recording occurred in a prospective manner (i.e., it is not likely that exposure misclassification rates might be differential by outcome status).

### Study base

The study base consisted of all patients with a primary TJR during the study period (CPRD: January 1, 1987 to August 31, 2012; DNHS: January 1, 1998 to December 31, 2007, the latest available data). We restricted TJR surgeries to procedures that were likely to be elective. All subjects were at least 40 years of age, had no record of hip or knee fracture in the previous 3 months, and had no history of rheumatoid arthritis. Patients were followed from the date of the primary TJR surgery to the end of data collection, the date of transfer of the patient out of the practice area (CPRD)/migration, the patient's death, or a revision TJR, whichever came first.

### Outcome of interest

The outcome of interest concerned implant revision surgery. In CPRD data, we identified revision surgery using CPRD Read codes. *International Classification Diseases, Tenth Edition*, procedure codes NFC and NGC were used to detect revision surgery in DNHS data.

### Cohort design

#### Time-dependent cohort study

**Figure 1.**
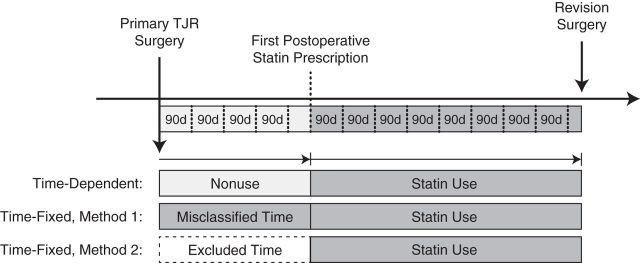
Overview of the 3 different cohort approaches used in this analysis of statin use and risk of implant revision surgery in the United Kingdom and Denmark, 1987–2012. Top row: time-dependent exposure status in which each patient may contribute to both “statin nonuse” and “statin use” groups. Middle row: time-fixed approach (method 1), (incorrectly) allocating immortal time before the first statin prescription to the statin use group (misclassification bias). Bottom row: time-fixed approach (method 2), excluding immortal time before the first statin prescription (selection bias). Medium gray shading represents statin use, light gray shading represents nonuse, and boxes with dashed borders represent excluded person-time. d, days; TJR, total joint replacement.

In this study design, statin exposure was defined in a time-dependent manner (see Figure 1). For statin users, total follow-up time was divided into 2 periods: 1) the first period started at the time of the primary TJR surgery and ended 1 day before the first postoperative statin prescription (this period was defined as nonuse), and 2) the second period started at the date of first postoperative statin prescription and ended at the end of follow-up. Individuals who were exposed to statins only before the primary TJR surgery were classified as nonusers. For statin nonusers, the total follow-up period was considered a period of nonuse.

#### Time-fixed immortal time cohort

For this design, the patient was defined as either a statin nonuser or a statin user and, consequently, could not move between these exposure statuses. Statin users were those with at least 1 statin prescription between the primary TJR surgery and the end of follow-up. All other patients were considered nonusers. Start of follow-up was defined as the date of the primary TJR surgery, regardless of exposure status.

#### Time-fixed exclusion cohort

This cohort design was similar to the time-fixed immortal time cohort design, with the exception of the start of follow-up. For statin users, the start of follow-up was defined as the date of the first postoperative statin prescription (i.e., the immortal time was excluded). For nonusers, the date of the primary TJR surgery remained the start of follow-up.

#### Case-control design

Nested within our study base, we selected all participants who underwent revision surgery, the date of which was considered the index date. For each case, up to 4 control individuals without revision surgery were selected using incidence density sampling. The index dates for the controls were imputed from the corresponding case. Statin exposure was defined as having at least 1 statin prescription between the date of the primary TJR and the index date.

### Potential confounders

The presence of risk factors was assessed at baseline—that is, on the date of the primary TJR surgery. Risk factors for implant failure were selected on the basis of their association with bone remodeling. These included age, sex, type of joint replaced (hip or knee), year of the primary TJR, and prior fractures. Further, we evaluated a history of comorbid diseases (osteoarthritis, inflammatory bowel disease, any malignancy, congestive heart failure, ischemic heart disease, cerebrovascular disease, rheumatoid arthritis, and chronic obstructive pulmonary disease) and use in the previous 6 months of medications that might affect bone modeling (bisphosphonates, calcium or vitamin D supplements, hormone replacement therapy, selective estrogen receptor modulators, glucose-lowering agents, proton pump inhibitors, antiarrhythmics, anticonvulsants, antidepressants, antiparkinsonian drugs, thiazide diuretics, and anxiolytics). For the CPRD population, we made additional adjustments for body mass index, smoking status, and alcohol use. All covariates (except age and body mass index) were treated as categorical variables, and missing information was treated as a separate category. Age and body mass index were handled as continuous variables (single linear terms).

As an additional step in the cohort studies, we further adjusted the analyses in a time-dependent manner. Total follow-up was divided into 90-day periods (Figure 1), and the confounder status was assessed at the start of each time interval.

### Statistical analysis

For the cohort designs, incidence rate ratios were estimated using Cox proportional hazards models (PHREG procedure; SAS, version 9.2, SAS Institute, Inc., Cary, North Carolina), comparing revision rates in statin users with those in nonusers. For the case-control design, we used logistic regression (LOGISTIC procedure; SAS, version 9.2). Conditional logistic regression was used for the propensity-matched case-control design. The hazard ratios from the cohort studies and the odds ratios from the case-control study are both estimates of the incidence rate ratio; hence, we expressed the risk estimates for all analyses as incidence rate ratios. We determined the influence of different confounder adjustment models:

1. No adjustments (crude)
2. Adjusted for age and sex
3. Additionally adjusted for comorbid diseases and drug use
4. Additionally adjusted for lifestyle parameters
5. Additionally adjusted for calendar time
6. Additionally adjusted for all potential confounders in a time-dependent fashion (this was considered the fully adjusted model)
7. Adjusted for all covariates that change the β coefficient for statin use by at least 1%, 5%, and 10% (change-in-estimate method). The change-in-estimate procedure was carried out evaluating each covariate on its own (comparing crude estimates with univariately adjusted estimates).
8. Propensity-adjusted model (including all potential confounders in the propensity model)
9. Propensity-matched model: In the cohort design, current statin users were matched to 1 nonuser by propensity score, modeled for statin use, with a maximum caliper width of 0.02 standard deviation; all potential confounders (assessed at the time of the primary TJR surgery) were included in the propensity model. In the case-control design, up to 4 controls were matched to each case using the same propensity score technique. Using this technique, we maintained incidence density sampling and were able to identify exactly 4 controls for each identified case.

To illustrate the dose-time-response relationship, we used smoothing spline regression to visualize the incidence rate ratio for revision surgery in relation to the cumulative number of statin daily defined doses after the primary TJR surgery (19–21).

#### Multicountry analysis

In order to evaluate regional differences, we conducted all of the above-mentioned analyses using the CPRD and DNHS databases separately, and accordingly, compared them against each other. Further, to aggregate the British and Danish results, we performed both an individual patient–level meta-analysis (lumping all individuals into a “mega-analysis”) and a classical meta-analysis (i.e., combining the incidence rate ratio estimates from the 2 separate data sources). When adjusting for potential confounders in the aggregation analysis, we considered only confounder data that were available in both databases. In the mega-analysis, we tested for multiplicative interaction (cross-product terms for statin use and database) as well as additive interaction between the 2 databases. For the classical meta-analysis, we used an inverse-variance fixed-effect design (Review Manager (RevMan), version 5.2; Nordic Cochrane Center (Cochrane Collaboration), Copenhagen, Denmark).

## RESULTS

We identified 119,182 British patients and 70,104 Danish patients who underwent primary TJR surgery (Table 1). Among these patients, 41.3% (*n* = 49,265) were classified as postoperative statin users in the British cohort, and 24.5% (*n* = 17,168) were classified as postoperative statin users in the Danish cohort. Baseline characteristics were very similar in the 2 data sources. Overall, statin users and nonusers had a similar mean age (approximately 70 years in British participants and approximately 68 years in Danish participants), and a higher proportion of statin users were males. We had a longer follow-up period in the British cohort (6.1 years for statin users and 5.2 years for nonusers) than in the Danish cohort (4.4 years for statin users and 3.9 years for nonusers). In both cohorts, statin users were more likely to have used glucose-lowering drugs and thiazide diuretics and more often had a history of ischemic heart disease, cerebrovascular disease, or hyperlipidemia. Among persons included in the cohorts, 3,517 British participants and 3,747 Danish participants underwent revision surgery. They were included in the case-control design and matched to 14,068 British and 14,988 Danish control subjects who did not undergo revision surgery.

**Table 1.** Baseline Characteristics of Statin Users and Nonusers, United Kingdom and Denmark, 1987–2012

Characteristic	United Kingdom (CPRD)				Denmark (DNHS)			
	Statin Use (*n* = 49,265),	Mean (SD)	Nonuse (*n* = 69,917),	Mean (SD)	Statin Use (*n* = 17,168),	Mean (SD)	Nonuse (*n* = 52,936),	Mean (SD)
	%		%		%		%	
Follow-up, years		6.1 (4.1)		5.2 (4.0)		4.4 (2.7)		3.9 (2.7)
Age at index date, years		70.2 (8.5)		69.5 (10.9)		68.2 (8.5)		68.3 (10.6)
Female sex	53.8		63.4		55.8		59.4	
Body mass index^a,b^		29.2 (5.1)		27.9 (5.3)				
Smoking status^c^								
Never smoker	54.3		61.7					
Current smoker	11.9		11.7					
Former smoker	33.7		24.2					
Alcohol use^d^								
No	21.4		19.2					
Yes	74.9		70.3					
Medication use within 6 months before index date								
Calcium or vitamin D	6.4		6.6		1.2		1.3	
Oral corticosteroids	4.9		5.0		8.4		8.1	
Noninsulin antidiabetics	12.0		2.1		14.1		2.9	
Thiazide diuretics	28.3		18.8		22.9		17.5	
Paracetamol or acetaminophen	62.5		56.4		33.8		30.4	
NSAIDs	52.0		52.6		62.0		61.6	
Opioids (tramadol or stronger)	37.1		34.8		29.4		28.4	
Bisphosphonates	4.7		4.7		2.3		2.7	
β blockers	25.4		10.7		23.8		11.1	
Antiplatelet drugs	37.7		11.2		35.4		13.3	
Anxiolytics or hypnotics	10.2		10.1		24.3		22.6	
Proton pump inhibitors	27.5		20.8		13.6		10.4	
Disease history before index date								
Fracture	20.7		20.5		21.6		24.0	
Osteoarthritis	76.4		72.0		97.8		97.4	
Rheumatoid arthritis	4.0		5.1		3.0		4.0	
Chronic kidney disease	8.9		4.5		1.0		0.7	
Heart failure	4.1		2.7		7.2		4.0	
Ischemic heart disease	24.9		5.7		23.8		6.3	
Cerebrovascular disease	9.9		3.6		7.2		2.9	
Hyperlipidemia	24.0		4.5		11.6		0.8	
Atrial fibrillation	5.1		3.3		6.6		4.2	
Hypertension	57.7		34.6		21.6		9.8	
Type 2 diabetes mellitus	15.5		2.5		10.6		2.2	
COPD	4.6		3.8		4.9		4.4	
Asthma	12.4		11.4		2.6		2.4	

Abbreviations: COPD, chronic obstructive pulmonary disease; CPRD, Clinical Practice Research Datalink; DNHS, Danish National Health System; NSAID, nonsteroidal antiinflammatory drug; SD, standard deviation.

^a^ Missing proportions in the CPRD: statin users, 2.7%; nonusers, 10.5%.

^b^ Calculated as weight (kg)/height (m)^2^.

^c^ Missing proportions in the CPRD: statin users, 0.1%; nonusers, 2.4%.

^d^ Missing proportions in the CPRD: statin users, 3.6%; nonusers, 10.5%.

**Figure 2.**
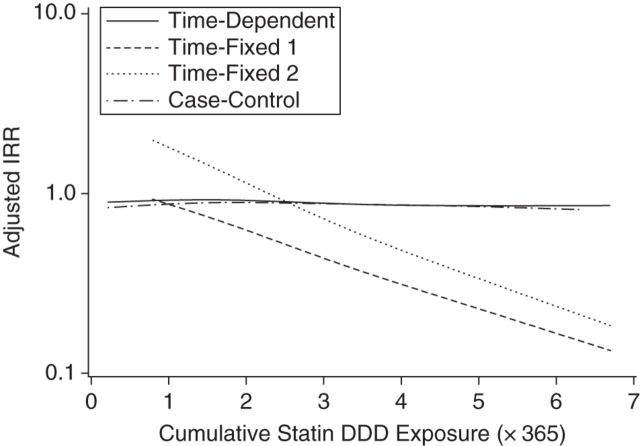
Spline regression plot of statin use and risk of implant revision surgery in relation to cumulative statin exposure (daily defined dose (DDD)), according to study design, United Kingdom, 1987–2012. Data from the Clinical Practice Research Datalink were used for this analysis. Yearly use was divided by the quantity corresponding to 1 DDD. The total number of DDDs used for statins during the study period was obtained as the sum of yearly DDDs. Results shown are from fully adjusted models. Time-fixed method 1 included misclassification of immortal time; time-fixed method 2 included exclusion of immortal time. IRR, incidence rate ratio.

Table 2 shows that there were no substantial differences between the results of the time-dependent cohort study and the case-control study. In British participants, the time-dependent cohort study yielded an adjusted incidence rate ratio of 0.92 (95% CI: 0.84, 1.01), closely resembling the risk estimate for the case-control study (adjusted incidence rate ratio (IRR) = 0.87, 95% CI: 0.79, 0.95). In contrast, both time-fixed cohort studies yielded substantially different results compared with the time-dependent cohort study and the case-control study (Table 2). Also in the British population, misclassification of immortal time resulted in an adjusted incidence rate ratio of 0.36 (95% CI: 0.33, 0.39). The association weakened, but risk remained substantially lower, when the period of time prior to starting a statin was excluded (adjusted IRR = 0.64, 95% CI: 0.58, 0.71). Both time-fixed approaches showed a significant relationship between the cumulative duration of statin use and the risk of revision surgery (Figure 2). This sharply contrasted with the time-dependent cohort study and the case-control study, which did not show such relationships. The same findings were observed in the Danish population.

**Table 2.** Risk of Orthopedic Implant Revision Surgery According to Statin Use, by Study Design, United Kingdom and Denmark, 1987–2012

Cohort Design	No Statin Use					Statin Use					
	IRR	No. of Events	No. of Person-Years	Rate per 10,000 Person-Years	Events, %^a^	IRR^b^	95% CI	No. of Events	No. of Person-Years	Rate per 10,000 Person-Years	Events, %^a^
*United Kingdom (CPRD)*											
Time-fixed (misclassification)	1	2,471	359,856	68.7	3.5	0.36	0.33, 0.39	1,046	299,640	34.9	2.1
Time-fixed (exclusion)	1	2,471	359,856	68.7	3.5	0.64	0.58, 0.71	1,046	210,395	49.7	2.1
Time-dependent	1	2,471	449,101	55.0	3.5	0.92	0.84, 1.01	1,046	210,395	49.7	2.1
Case-control	1	2,471				0.87	0.79, 0.95	1,046			
*Denmark (DNHS)*											
Time-fixed (misclassification)	1	3,220	207,154	155.4	6.1	0.36	0.33, 0.40	527	74,655	70.6	3.1
Time-fixed (exclusion)	1	3,220	207,154	155.4	6.1	0.65	0.59, 0.72	527	42,510	124.0	3.1
Time-dependent	1	3,220	239,298	134.6	6.1	0.90	0.81, 0.99	527	42,510	124.0	3.1
Case-control	1	3,220				0.85	0.76, 0.95	527			

Abbreviations: CI, confidence interval; CPRD, Clinical Practice Research Datalink; DNHS, Danish National Health System; IRR, incidence rate ratio.

^a^ Percentage of patients at risk who had events.

^b^ Adjusted for age, sex, type of replaced joint, year of the primary surgery, history of fracture, comorbid diseases (osteoarthritis, inflammatory bowel disease, any malignancy, congestive heart failure, ischemic heart disease, cerebrovascular disease, rheumatoid arthritis, and chronic obstructive pulmonary disease), and medication use (bisphosphonates, calcium or vitamin D supplements, hormone replacement therapy, selective estrogen receptor modulators, glucose-lowering agents, proton pump inhibitors, antiarrhythmics, anticonvulsants, antidepressants, antiparkinsonian drugs, thiazide diuretics, and anxiolytics). With CPRD data, results were additionally adjusted for body mass index, smoking status, and alcohol use.

There were no substantial differences between the various techniques for dealing with confounding (Table 3). For example, in the British cohort, using a time-dependent approach, the fully adjusted incidence rate ratio was 0.92 (95% CI: 0.84, 1.01). The incidence rate ratio was 0.92 (95% CI: 0.84, 1.01) with the change-in-estimate method (1% change), 0.96 (95% CI: 0.88, 1.04) using propensity-adjusted models, and 0.96 (95% CI: 0.87, 1.05) with the propensity-matched models. The same trend was present in the Danish analyses (data not shown).

**Table 3.** Risk of Orthopedic Implant Revision Surgery Among Statin Users^a^ According to Method Used to Deal With Confounding, Clinical Practice Research Datalink, United Kingdom, 1987–2012

Confounder-Handling Technique	Cohort Design						Case-Control Design	
	Time-Fixed				Time-Dependent			
	Method 1^b^		Method 2^c^					
	IRR	95% CI	IRR	95% CI	IRR	95% CI	IRR	95% CI
Unadjusted^d^	0.51	0.47, 0.55	0.72	0.67, 0.78	0.90	0.84, 0.97	0.89	0.82, 0.95
Cox proportional hazards regression								
Adjustments (at baseline only)								
Age and sex	0.50	0.46, 0.54	0.73	0.67, 0.78	0.93	0.86, 1.00	0.89	0.83, 0.96
Plus comorbid diseases or drug use^e^	0.42	0.39, 0.46	0.67	0.61, 0.73	0.90	0.83, 0.98	0.88	0.81, 0.97
Plus lifestyle factors^f^	0.42	0.38, 0.45	0.65	0.60, 0.71	0.90	0.82, 0.97	0.88	0.80, 0.96
Plus calendar time	0.42	0.38, 0.45	0.66	0.61, 0.72	0.91	0.84, 0.99	0.87	0.79, 0.95
Plus time-dependent adjustment	0.36	0.33, 0.39	0.64	0.58, 0.71	0.92	0.84, 1.01		
Change-in-estimate method								
>1% change^g^	0.43	0.40, 0.47	0.67	0.62, 0.73	0.92	0.84, 1.01	0.86	0.79, 0.95
>5% change^h^	0.45	0.41, 0.48	0.67	0.62, 0.73	0.92	0.84, 1.01	0.86	0.78, 0.95
>10% change^i^	0.51	0.47, 0.55	0.72	0.67, 0.78	0.96	0.88, 1.04	0.89	0.83, 0.96
Propensity score^j^	0.44	0.40, 0.47	0.67	0.61, 0.73	0.96	0.88, 1.04	0.85	0.78, 0.94
Propensity-matched^j^	0.41	0.37, 0.45	0.65	0.59, 0.71	0.96	0.87, 1.05	0.85	0.77, 0.95

Abbreviations: CI, confidence interval; IRR, incidence rate ratio.

^a^ Referent: no statin use (IRR = 1).

^b^ Misclassification of immortal time.

^c^ Exclusion of immortal time.

^d^ Not adjusted for any of the potential confounders, including age and sex.

^e^ Adjusted for age, sex, type of replaced joint, year of the primary surgery, history of fracture, comorbid diseases (osteoarthritis, inflammatory bowel disease, any malignancy, congestive heart failure, ischemic heart disease, cerebrovascular disease, rheumatoid arthritis, and chronic obstructive pulmonary disease), and drug use (bisphosphonates, calcium or vitamin D supplements, hormone replacement therapy, selective estrogen receptor modulators, glucose-lowering agents, proton pump inhibitors, antiarrhythmics, anticonvulsants, antidepressants, antiparkinsonian drugs, thiazide diuretics, and anxiolytics).

^f^ Adjusted for variables listed in footnote e and for lifestyle factors (body mass index, smoking status, alcohol use, and general practitioner deprivation score (25)).

^g^ Cohort design: adjusted for age, sex, type of replaced joint, year of the primary surgery, use of thiazide diuretics, a history of cerebrovascular disease, body mass index, and smoking status. Case-control design: adjusted for age, sex, use of proton pump inhibitors, a history of cerebrovascular disease, body mass index, and smoking.

^h^ Cohort design: adjusted for variables listed in footnote g and for the use of glucose-lowering drugs, proton pump inhibitors, and antidepressants. Case-control design: adjusted for variables listed in footnote g and for alcohol use, congestive heart failure, ischemic heart disease, and the use of glucose-lowering drugs, anticonvulsants, and antidepressants.

^i^ Cohort design: adjusted for variables listed in footnote g and for hormone replacement therapy, antiparkinsonian drugs, congestive heart failure, ischemic heart disease, and chronic obstructive pulmonary disease. Case-control design: adjusted for variables listed in footnote g and for alcohol use, use of glucose-lowering drugs, antiarrhythmics, thiazide diuretics, and anxiolytics, history of fracture, and chronic obstructive pulmonary disease.

^j^ Propensity model (outcome = statin use) included all potential confounders and yielded a *C* statistic score of 0.80 for all 3 cohort designs and 0.89 for the case-control design.

In a sensitivity analysis, we imputed the missing data, but this did not substantially alter our findings. For example, in the CPRD data set, modeling the missing data category yielded an adjusted incidence rate ratio of 0.92 (95% CI: 0.84, 1.01) as compared with an adjusted incidence rate ratio of 0.94 (95% CI: 0.85, 1.04) when missing data were imputed. Both estimates were obtained using the fully adjusted time-dependent model.

Table 4 shows the results of aggregating the British and Danish findings. Using the time-dependent approach, the individual patient–level mega-analysis yielded an adjusted incidence rate ratio of 0.90 (95% CI: 0.85, 0.96), which was comparable to the result of the classical meta-analysis (adjusted IRR = 0.91, 95% CI: 0.86, 0.98). No heterogeneity was present in the classical meta-analysis (*P* = 0.78, *I*^2^ = 0%). There was no statistical multiplicative interaction with the data source in the individual patient–level mega-analysis (*P* = 0.46), nor could we observe statistically significant additive interaction. Further stratification of the study period to the “statin era” (January 2000 to August 2012) did not diminish the observed modestly decreased risk of revision surgery (adjusted IRR = 0.91, 95% CI: 0.83, 0.99).

**Table 4.** Risk of Orthopedic Implant Revision Surgery Among Statin Users^a^ in British and Danish Data Sets, Separately and in Aggregate, United Kingdom and Denmark, 1987–2012

Adjustment and Data Set	Cohort Design						Case-Control Design	
	Time-Fixed				Time-Dependent			
	Method 1^b^		Method 2^c^					
	IRR	95% CI	IRR	95% CI	IRR	95% CI	IRR	95% CI
Unadjusted^d^								
United Kingdom (CPRD)	0.51	0.47, 0.55	0.72	0.67, 0.78	0.90	0.84, 0.97	0.89	0.82, 0.95
Denmark (DNHS)	0.45	0.41, 0.50	0.80	0.73, 0.88	0.92	0.84, 1.01	0.92	0.83, 1.00
Meta-analysis	0.49	0.46, 0.52	0.75	0.71, 0.80	0.91	0.86, 0.96	0.90	0.85, 0.95
Mega-analysis	0.49	0.46, 0.52	0.75	0.71, 0.80	0.91	0.86, 0.96	0.90	0.85, 0.95
Age- and sex-adjusted								
United Kingdom (CPRD)	0.50	0.46, 0.54	0.73	0.67, 0.78	0.93	0.86, 1.00	0.89	0.83, 0.96
Denmark (DNHS)	0.45	0.41, 0.49	0.80	0.73, 0.87	0.93	0.85, 1.02	0.92	0.84, 1.01
Meta-analysis	0.48	0.45, 0.51	0.75	0.71, 0.80	0.93	0.88, 0.99	0.90	0.85, 0.96
Mega-analysis	0.48	0.45, 0.51	0.75	0.71, 0.80	0.93	0.88, 0.99	0.91	0.86, 0.96
Fully adjusted^e^								
United Kingdom (CPRD)	0.36	0.33, 0.39	0.64	0.58, 0.71	0.92	0.84, 1.01	0.87	0.79, 0.95
Denmark (DNHS)	0.36	0.33, 0.40	0.65	0.59, 0.72	0.90	0.81, 0.99	0.85	0.76, 0.95
Meta-analysis	0.36	0.34, 0.38	0.65	0.63, 0.68	0.91	0.86, 0.98	0.86	0.80, 0.92
Mega-analysis^f^	0.36	0.34, 0.38	0.65	0.63, 0.68	0.90	0.85, 0.96	0.87	0.81, 0.93

Abbreviations: CI, confidence interval; CPRD, Clinical Practice Research Datalink; DNHS, Danish National Health System; IRR, incidence rate ratio.

^a^ Referent: no statin use (IRR = 1).

^b^ Misclassification of immortal time.

^c^ Exclusion of immortal time.

^d^ Not adjusted for any of the potential confounders, including age and sex.

^e^ Adjusted for confounders shown in Table 2.

^f^ Adjusted for confounders shown in Table 2, excluding body mass index, smoking status, and alcohol use.

## DISCUSSION

This study shows that there is probably no causal relationship between statins and implant failure in patients with a replaced hip or knee. Both the case-control design (IRR = 0.87) and the time-dependent cohort design (IRR = 0.90) revealed a slight reduction in implant failure rates. However, the risk did not decrease with a longer duration of statin use. The time-fixed cohort analyses led to substantially lower risk estimates; this observation held for both time-fixed cohort designs but was greater when immortal time was misclassified (IRR = 0.36) than when it was excluded (IRR = 0.65). Differences in confounder-handling techniques or data sources did not substantially alter the study findings.

In line with our findings, Thillemann et al. (9) found an overall protective association of statins with implant failure, with the absence of a clear dose-response relationship, in a Danish case-control study. However, the magnitude of their observed association (IRR = 0.34) was substantially greater than the magnitudes of any of the incidence rate ratios we found in our Danish case-control designs (lowest IRR = 0.85). In contrast to the first Danish study (9), we did not use dedicated joint replacement registries to identify revision surgery. Theoretically, this might have led to underrecording of the outcome in our study. The revision rate observed in our study, however, was not lower than that in the first Danish study. Moreover, this underrecording is likely to have been nondifferential, and it is likely that the databases record revision surgery with near-perfect specificity. Therefore, underrecording should not have had a major influence on the incidence rate ratios.

There is conflicting evidence on the beneficial effects of statins on bone metabolism. In vitro and in vivo, statins inhibit osteoclast-mediated bone resorption, by reducing mevalonic acid formation (12). However, randomized controlled trials could not confirm this potential benefit on fracture risk (14). While some observational studies suggested a protective effect for statins in preventing fracture (7), others could not find such an association (13).

The difference between the time-fixed and time-dependent cohort designs emphasizes the importance of immortal time bias. In time-fixed cohorts, the immortal time can be either misclassified or excluded (Figure 1). Both may lead to substantial bias, as was shown in our study. Misclassification of immortal time results in lengthened person-time among statin users and shortened person-time among nonusers (both resulting in a downward bias). Exclusion of immortal time shortens person-time only among nonusers, which results in a downward bias as well, albeit less pronounced than when immortal time is misclassified. Time-dependent cohort or nested case-control designs are appropriate methods for preventing immortal time bias (22). Because case-control designs do not take person-time into account, there is no misclassification or selection of immortal time bias by definition. Our example showed that these 2 designs were comparable in terms of the overall association as well as in the duration-of-use analysis.

The various methods for dealing with confounding did not produce differences in the rate ratios. This is in line with a simulation study comparing various propensity score methods with conventional regression adjustment (23). In this simulation study, propensity adjustment, propensity matching, and conventional regression adjustment were comparable in terms of bias reduction and precision. However, propensity scores perform better when a large number of prognostic factors are included in the regression model, the treatment effect becomes larger, and the incidence of the outcome increases (24). It seems that these differences are based on artifacts in the conventional regression model under these circumstances rather than on a better handling of confounding by indication or residual confounding. Interestingly, the magnitude of immortal time bias became larger with more intense modeling. This could be explained by unhealthy bias (adjusting for unhealthy status among statin users may amplify the immortal time bias) or modeling artifacts (24).

The use of different data sources was not a discriminating factor in this example of statins and implant revision. Although the British and Danish electronic health records differ in some important aspects, the results in the incidence rate ratio analyses were comparable. Absolute revision rates were substantially higher in the Danish population than in British individuals. This was probably the result of underrecording using general practice electronic health records.

Strengths of this study included a large sample size, longitudinal follow-up, use of data sources from multiple countries, the ability to compare different study designs, and the availability of routinely collected data on exposure, outcome, and potential confounders. The routine data collection allowed us to analyze very fine timing patterns with the precision of a single day. A major limitation of our study is that we used a proxy measure for implant failure—revision surgery. Revision surgery after implant failure may be conditional on surgical fitness, and this may have distorted our study findings. Furthermore, statin use—in particular, adherence to statin use—may be associated with healthy user bias. There is a chance that revision surgery was underrecorded, but this is likely to have been nondifferential. When using the case-control design, we were not able to ensure that recording of data on covariates preceded exposure. It may be postulated that, ideally, data recording should precede exposure, even in a case-control design. However, our primary objective was to mimic widely used methodologies. Because in most case-control studies, covariates are assessed prior to the index date (here, the date of revision surgery) rather than prior to ascertainment of exposure status, we aimed to mimic this methodology as much as possible. Moreover, it is difficult to ensure that data recording precedes exposure, in particular when the case or control patient is “unexposed” (in case-control designs, it is then unclear what the date of exposure ascertainment should be).

In conclusion, statins probably do not prevent implant failure in individuals with a TJR surgery. Despite a small inverse association, the duration-of-use analysis did not support a causal relationship. The time-fixed cohort designs resulted in a substantially biased risk estimate, regardless of the type of time-fixed design. Confounder-handling techniques (including propensity score analyses) and type of electronic health record seemed to play only a minor role in differences in study findings. When the association between statin use and revisions after TJR is evaluated, conventional study designs such as case-control designs or cohort designs that take into account time-varying aspects of exposure are better than time-fixed cohort study designs. From a clinical perspective, this study does not support the use of statins to prevent joint implant failure in patients who underwent primary total hip or knee replacement surgery.

## References

[1] Harris WH, Sledge CB Total hip and total knee replacement (1). *N Engl J Med*. 1990;323 11:725–731. 2201916 10.1056/NEJM199009133231106

[2] Agency for Healthcare Research and Quality. *Healthcare Cost and Utilization Project 2009*. Rockville, MD: Agency for Healthcare Research and Quality; 2009.

[3] Datamonitor Healthcare. *Datamonitor Healthcare Report: Hip and Knee Replacement Market: Product Code: DMHC2264*. London, United Kingdom: Datamonitor Healthcare; 2006.

[4] Havelin LI, Fenstad AM, Salomonsson R et al The Nordic Arthroplasty Register Association: a unique collaboration between 3 national hip arthroplasty registries with 280,201 THRs. *Acta Orthop*. 2009;80 4:393–401. 19513887 10.3109/17453670903039544 PMC2823198

[5] de Steiger RN, Miller LN, Prosser GH et al Poor outcome of revised resurfacing hip arthroplasty. *Acta Orthop*. 2010;81 1:72–76. 20170416 10.3109/17453671003667176 PMC2856207

[6] Hossain F, Patel S, Haddad FS Midterm assessment of causes and results of revision total knee arthroplasty. *Clin Orthop Relat Res*. 2010;468 5:1221–1228. 20058112 10.1007/s11999-009-1204-0 PMC2853653

[7] Meier CR, Schlienger RG, Kraenzlin ME et al HMG-CoA reductase inhibitors and the risk of fractures. *JAMA*. 2000;283 24:3205–3210. 10866867 10.1001/jama.283.24.3205

[8] Nielsen SF, Nordestgaard BG, Bojesen SE Statin use and reduced cancer-related mortality. *N Engl J Med*. 2012;367 19:1792–1802. 23134381 10.1056/NEJMoa1201735

[9] Thillemann TM, Pedersen AB, Mehnert F et al The risk of revision after primary total hip arthroplasty among statin users: a nationwide population-based nested case-control study. *J Bone Joint Surg Am*. 2010;92 5:1063–1072. 20439650 10.2106/JBJS.H.01805

[10] Staal A, Frith JC, French MH et al The ability of statins to inhibit bone resorption is directly related to their inhibitory effect on HMG-CoA reductase activity. *J Bone Miner Res*. 2003;18 1:88–96. 12510809 10.1359/jbmr.2003.18.1.88

[11] von Knoch F, Wedemeyer C, Heckelei A et al Promotion of bone formation by simvastatin in polyethylene particle-induced osteolysis. *Biomaterials*. 2005;26 29:5783–5789. 15869791 10.1016/j.biomaterials.2005.02.008

[12] Laing AJ, Dillon JP, Mulhall KJ et al Statins attenuate polymethylmethacrylate-mediated monocyte activation. *Acta Orthop*. 2008;79 1:134–140. 18283585 10.1080/17453670710014888

[13] van Staa TP, Wegman S, de Vries F et al Use of statins and risk of fractures. *JAMA*. 2001;285 14:1850–1855. 11308398 10.1001/jama.285.14.1850

[14] de Vries F, de Vries C, Cooper C et al Reanalysis of two studies with contrasting results on the association between statin use and fracture risk: the General Practice Research Database. *Int J Epidemiol*. 2006;35 5:1301–1308. 17053011 10.1093/ije/dyl147

[15] Prieto-Alhambra D, Javaid MK, Judge A et al Association between bisphosphonate use and implant survival after primary total arthroplasty of the knee or hip: population based retrospective cohort study. *BMJ*. 2011;343:d7222. 22147909 10.1136/bmj.d7222 PMC3232250

[16] Thillemann TM, Pedersen AB, Mehnert F et al Postoperative use of bisphosphonates and risk of revision after primary total hip arthroplasty: a nationwide population-based study. *Bone*. 2010;46 4:946–951. 20102756 10.1016/j.bone.2010.01.377

[17] Suissa S Immortal time bias in observational studies of drug effects. *Pharmacoepidemiol Drug Saf*. 2007;16 3:241–249. 17252614 10.1002/pds.1357

[18] Bazelier MT, de Vries F, Vestergaard P et al Risk of fracture with thiazolidinediones: an individual patient data meta-analysis. *Front Endocrinol (Lausanne)*. 2013;4:11. 23549934 10.3389/fendo.2013.00011 PMC3582108

[19] Lalmohamed A, Vestergaard P, Klop C et al Timing of acute myocardial infarction in patients undergoing total hip or knee replacement: a nationwide cohort study. *Arch Intern Med*. 2012;172 16:1229–1235. 22826107 10.1001/archinternmed.2012.2713

[20] Lalmohamed A, Vestergaard P, Cooper C et al Timing of stroke in patients undergoing total hip replacement and matched controls: a nationwide cohort study. *Stroke*. 2012;43 12:3225–3229. 23132782 10.1161/STROKEAHA.112.668509

[21] Lalmohamed A, Vestergaard P, Jansen PA et al Prolonged outpatient vitamin K antagonist use and risk of venous thromboembolism in patients undergoing total hip or knee replacement. *J Thromb Haemost*. 2013;11 4:642–650. 23387806 10.1111/jth.12158

[22] Lévesque LE, Hanley JA, Kezouh A et al Problem of immortal time bias in cohort studies: example using statins for preventing progression of diabetes. *BMJ*. 2010;340:b5087. 20228141 10.1136/bmj.b5087

[23] Austin PC The performance of different propensity-score methods for estimating relative risks. *J Clin Epidemiol*. 2008;61 6:537–545. 18471657 10.1016/j.jclinepi.2007.07.011

[24] Martens EP, Pestman WR, de Boer A et al Systematic differences in treatment effect estimates between propensity score methods and logistic regression. *Int J Epidemiol*. 2008;37 5:1142–1147. 18453634 10.1093/ije/dyn079

[25] Herrett E, Gallagher AM, Bhaskaran K et al Data resource profile: Clinical Practice Research Datalink (CPRD). *Int J Epidemiol*. 2015;44 3:827–836. 26050254 10.1093/ije/dyv098 PMC4521131

